# Topoisomerase poisoning by the flavonoid nevadensin triggers DNA damage and apoptosis in human colon carcinoma HT29 cells

**DOI:** 10.1007/s00204-021-03162-5

**Published:** 2021-10-12

**Authors:** Lena Müller, Larissa Rhonda Friederike Schütte, David Bücksteeg, Julian Alfke, Thomas Uebel, Melanie Esselen

**Affiliations:** Institute of Food Chemistry, University of Münster, Corrensstraße 45, 48149 Münster, Germany

**Keywords:** Plant polyphenol, Flavone, Topoisomerase I, Caspase activity, Cell cycle arrest

## Abstract

Nevadensin, an abundant polyphenol of basil, is reported to reduce alkenylbenzene DNA adduct formation. Furthermore, it has a wide spectrum of further pharmacological properties. The presented study focuses the impact of nevadensin on topoisomerases (TOPO) in vitro. Considering the DNA-intercalating properties of flavonoids, first, minor groove binding properties (IC_50_ = 31.63 µM), as well as DNA intercalation (IC_50_ = 296.91 µM) of nevadensin, was found. To determine potential in vitro effects on TOPO I and TOPO IIα, the relaxation and decatenation assay was performed in a concentration range of 1–500 µM nevadensin. A partial inhibition was detected for TOPO I at concentrations  ≥ 100 µM, whereas TOPO IIα activity is only inhibited at concentrations  ≥ 250 µM. To clarify the mode of action, the isolating in vivo complex of enzyme assay was carried out using human colon carcinoma HT29 cells. After 1 h of incubation, the amount of TOPO I linked to DNA was significantly increased by nevadensin (500 µM), why nevadensin was characterized as TOPO I poison. However, no effects on TOPO IIα were detected in the cellular test system. As a subsequent cellular response to TOPO I poisoning, a highly significant increase of DNA damage after 2 h and a decrease of cell viability after 48 h at the same concentration range were found. Furthermore, after 24 h of incubation a G_2_/M arrest was observed at concentrations ≥ 100 µM by flow cytometry. The analysis of cell death revealed that nevadensin induces the intrinsic apoptotic pathway via activation of caspase-9 and caspase-3. The results suggest that cell cycle disruption and apoptotic events play key roles in the cellular response to TOPO I poisoning caused by nevadensin in HT29 cells.

**Supplementary Information:**

The online version contains supplementary material available at 10.1007/s00204-021-03162-5.

## Introduction

**Fig. 1:**
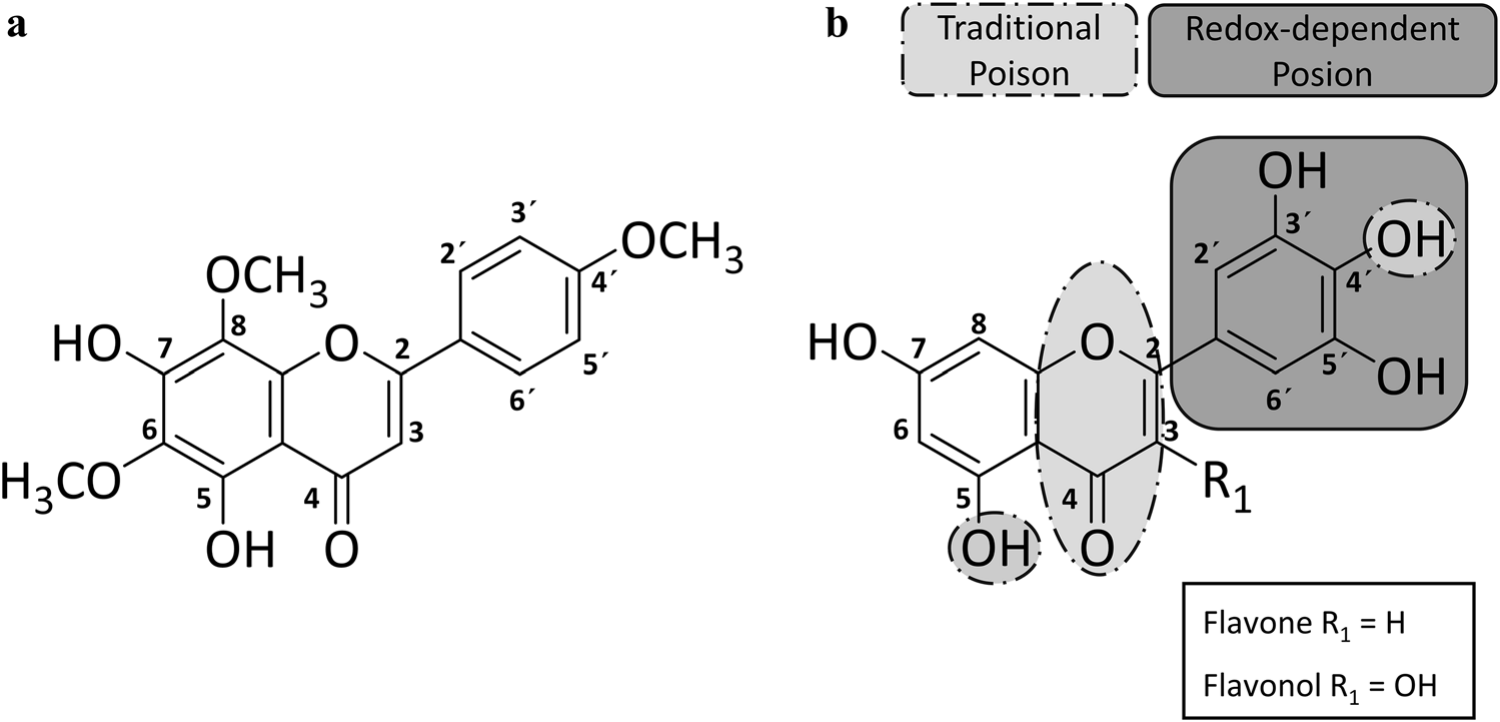
Chemical structure of nevadensin (**a**). Crucial structure criteria for flavonoids acting as traditional and redox-dependent topoisomerase poisons (**b**) modified according to Bandele et al. (2008). Redox-dependent poisoning is associated with DNA-binding affinity and oxidative transformation to quinones. In the case of traditional poisons, the hydroxy groups in positions C-5 and C-4′, as well as the aromatic, planar C ring with a ketone group in position C-4 are identified key elements

Flavonoids are widely distributed throughout the plant kingdom and exhibit a high structural diversity. Daily dietary intake of mixed flavonoids is determined in the human population and ranges from 167 to 564 mg per day, respectively (Wisnuwardani et al. 2019). Furthermore, this substance class is most commonly found in commercially available high-dose supplements with a daily recommended dose up to 2 g per day (Espín et al. 2007). For example, human plasma concentrations about 52 nM of the abundant flavonol quercetin has been reported after normal dietary intake (Erlund et al. 2002). Flavonoid-associated health benefits comprise, for example, anti-inflammatory and anti-oxidative activities as well as protection against cardiovascular diseases (Ross and Kasum 200). Nevadensin (Fig. 1a), 5,7-dihydroxy-6,8,4′-trimethoxyflavone (CAS No.: 10176-66-6) belongs to the class of secondary plant metabolites. It is mainly found in *Ocimum basicilum* L. and *Ocimum americanum* L. as well as in many other plants, such as *Helianthus pumilus* N., *Lysionotus pauciflorus* M. and *Cheilanthes argentea* F. (Berim and Gang 2016; Herz and Groote 1977). The concentrations of nevadensin are indicated between 0.6 and 14.6 mg/kg dry weight in basil (Grayer et al. 2001). Moreover, the quantity of nevadensin in basil-containing plant food supplements is reported to be in the range of 0.14–0.24 mg/g supplement (van den Berg et al. 2013).

To date, numerous studies highlight the bioactive properties of nevadensin, e.g. anti-inflammatory effects, anti-tumour and anti-carcinogenic activity, hypotensive activity, anti-tubercular, and anti-microbial activity (Brahmachari 2010). Furthermore, direct enzyme-modulating effects of flavonoids are already demonstrated in general as well as for nevadensin itself. Nevadensin is identified as a selective inhibitor of human carboxylesterase 1 by Wang and co-workers (2018). Moreover, nevadensin potently inhibits p40 protein tyrosine kinase activity with an IC_50_ value of 50 μg/mL. Additionally, different in vitro and in vivo studies show that nevadensin reduces the amount of alkenylbenzenes-specific DNA adducts catalysed by sulfotransferases (Alhusainy et al. 2010). It should be noted that enzyme-modulating properties of flavonoids are also controversially discussed in literature (Miron et al. 2017). Thereby, the enzyme class of topoisomerases (TOPO) is particularly of interest. TOPOs are nuclear phosphor proteins, ubiquitously occuring in eukaryotic as well as in prokaryotic cells. These enzymes are essential for nuclear processes that are strongly dependent on changes in DNA topology (Baranello et al. 2013). Changes in the topological arrangement are induced by relaxing torsional stress in supercoiled DNA sectors during initial steps of replication, transcription, and recombination (Baranello et al. 2013). TOPOs are classified into two main classes: type I and type II, which differ between their structure and their corresponding mode of action (Champoux 2001). Enzymes, which cleave single-strand breaks in DNA double helix are defined as topoisomerase I (TOPO I). The isoforms that cleave ATP-dependent double-strand breaks are known as topoisomerase II (TOPO II) (Deweese and Osheroff 2010). The catalytic cycles of both enzyme classes exhibit a fixed and characteristic order of successive steps and depends on contacting and detaching sequences between enzyme and double-strand DNA. The initial phase of the cycle is the generation of an incision in the DNA, also called nicking. Consequently, the DNA backbone breaks, and the DNA is attached to the enzyme. Subsequently, the DNA is rearranged in an unwound topology before closing the strand break. Finally, the catalytic cycle results in unwounded DNA and the release of the enzyme from the cleavage complex (Champoux 2001). This catalytic cycle is not an unregulated and autonomous pathway, so drugs can interact with the cleavage complex or specifically intercalate into the processed DNA at any level of the TOPO cycle.

Xenobiotics which influence TOPO activity are divided into two classes: the so-called TOPO poisons and the catalytic TOPO inhibitors. Fundamentally, poisons and inhibitors differ in their mode of action, whereby the poisons stabilise the enzyme/DNA complex, and the inhibitors interrupt the catalytic cycle of the enzyme before DNA-binding (Beretta et al. 2012, 2008). Both mechanisms result in an imbalance between enzyme/DNA complexes and free enzyme. Permanent DNA strand breaks above or below a critical level of enzyme/DNA complexes are induced, which cause the activation of stress-associated signalling pathways, e.g. cell cycle arrest or apoptosis induction by caspases activation. The effect intensity of TOPO inhibitors is roughly proportional to the cellular enzyme content. Accordingly, the DNA-damaging effect increase with higher TOPO concentrations. In fast proliferating malignant cells TOPO enzymes are highly expressed. Therefore, these cells particularly respond to TOPO-inhibiting effects (Beretta et al. 2012, 2008; Fry et al. 1991).

For this reason, TOPO poisons as well as catalytic inhibitors are attractive candidates for a clinical use as anti-cancer agents. Camptothecin (CPT) and its analogues topotecan, irinotecan, and namitecan are well-known TOPO I poisons. Further common cytostatic agents are TOPO II poisons such as the anthracycline doxorubicin or the podophyllotoxin etoposide (ETP) (Hevener et al. 2018). Besides these therapeutics, it is already known that a large number of food-borne compounds can strongly influence TOPO activity, which is exemplarily reviewed by Esselen and Barth (2014).

Different structural characteristics of flavonoids are discussed to be essential for the inhibition of the TOPO-mediated DNA relegation step (López-Lázaro et al. 2010; Mittra et al. 2000; Yamashita and Kawanishi 2000). In literature, they are classified as redox-dependent poisons (Fig. 1b; marked dark-grey) and traditional poisons (Fig. 1b, marked grey).

Due to the ubiquitous occurrence of flavonoids in food and furthermore in the expanded market of highly enriched and microencapsulated dietary supplements, an assessment of possible application restrictions of these compounds is of high relevance. So far, there are no studies on potential accumulative effects, but the consumption of natural TOPO poisons among the group of flavonoids during pregnancy is already associated with the clinical picture of infantile leukaemia (Spector et al. 2005; Strick et al. 2000).

This present study addressed the question whether the natural plant flavonoid nevadensin acts as a potential topoisomerase poison in vitro. Furthermore, the potential cellular responses including cell cycle distribution and apoptosis induction are investigated.

## Materials and methods

### Chemicals

HT29 cell line (DSMZ Cat# ACC-299, RRID: CVCL_0320) was obtained from Leibnitz Institute DSMZ (Braunschweig, Germany). Nevadensin (CAS number: 10176-66-6, purity: > 99%) was purchased from Biosynth Carbosynth (Berkshire, United Kingdom) and dimethyl sulfoxide (DMSO) from Carl Roth GmbH & Co. KG (Karlsruhe, Germany). All other chemicals were of the highest commercially available quality purchased from selected suppliers.

### Cell culture

HT29 cells were cultured in Dulbecco's Modified Eagle's Medium (Gibco®, Thermo Fisher Scientific BV & Co. KG, Braunschweig, Germany) supplemented with fetal calf serum (FCS; 10%, Gibco®, Thermo Fisher Scientific BV & Co. KG, Braunschweig, Germany) and penicillin/streptomycin (1%, Gibco®, Thermo Fisher Scientific BV & Co. KG, Braunschweig, Germany) at 37 °C, 5% CO_2_ and relative humidity. The same conditions were used for all cell culture experiments. All cell culture experiments were carried out with an addition of 100 U/mL catalase (Sigma-Aldrich GmbH, Seelze, Germany) to prevent the formation of hydrogen peroxide from nevadensin, widely reported for many flavonoids (Kern et al. 2007; Fritz et al. 2008; Long et al. 2000; Chai et al. 2003a, b). Furthermore, all cell culture experiments were carried out with nevadensin in a concentration range of 1–500 µM. DMSO (1%) was used as negative control (NC) whereas camptothecin (CPT) served as positive control (PC) in all in vitro assays, excluding DNA-intercalation assays and LDH assay. The CPT concentration was adjusted to the respective assay. Etoposide (ETP) served as positive control for decatenation assay and ICE-assay.

### DNA binding properties

DNA-binding properties were measured according to the protocol of Morgen and co-workers (1979) modified by Habermeyer and co-workers (2005) in 96-well plates (Sarstedt, Nümbrecht, Germany). A decrease in fluorescence intensity resulted from the replacement of the intercalator ethidium bromide (1 μM; Sigma-Aldrich GmbH, Seelze, Germany) in double-stranded DNA (calf thymus DNA; Sigma-Aldrich GmbH, Seelze, Germany) or the minor-groove binder Hoechst 33258 (1 µM; Sigma-Aldrich GmbH, Seelze, Germany). Fluorescence was measured at *λ*_ex_ at 544 nm and *λ*_em_ at 590 nm or at *λ*_ex_ of 355 nm and *λ*_em_ of 460 nm with an Infinite M200 PRO microplate reader (Tecan Group Ltd., Männedorf, Switzerland), respectively.

### Relaxation assay

Relaxation assay was carried out according to the protocol of Habermeyer and co-workers (2005). Effects of nevadensin on human TOPO I activity were investigated by the unwinding of supercoiled pUC18 DNA into its relaxed form. Plasmid DNA (250 ng pUC18) was incubated in a solution containing 1 μL of TOPO I (nuclear extract of MCF-7 cells); 100 μM Tris–HCl; pH 7.9; 1 M KCl; 100 mM MgCl_2_; 5 mM DTT; 5 mM EDTA; and 0.3 mg/mL bovine serum albumin (BSA) for 30 min at 37 °C. Reactions were stopped with 5% SDS solution (w/v), and proteinase K solution (10 mg/mL) was added. After that, incubation was repeated for 30 min at 37 °C. Gel electrophoresis was carried out at 60 V for 3 h in an agarose gel 1%, (w/v) with Tris–acetate/EDTA (TAE) buffer (20 mL TAE stock solution: 242 g Tris, 100 mL EDTA (500 mM, pH 8), 57.1 mL acetic acid (99%); ad 980 mL H_2_O). Finally, the gel was stained with ethidium bromide (10 μg/mL) for 20 min and detected with a ChemiDoc XRS system (Biorad, München, Germany).

### Decatenation assay

Decatenation assay was performed to measure effects on the activity of TOPO II according to the protocol of Habermeyer and co-workers (2005). Catenated kinetoplast DNA (kDNA) (330 ng, TopoGEN, Ohio, US) was used as substrate for TOPO II. The assay was performed in a volume of 30 µL, containing 40 ng of TOPO II (TopoGEN, Ohio, US); 50 mM Tris, pH 7.9; 120 mM KCl; 10 mM MgCl_2_; 1 mM ATP; 0.5 mM DTT; 0.5 mM EDTA; and 0.03 mg/mL BSA. Reaction mixtures were incubated at 37 °C for 60 min. Reactions were stopped by adding proteinase K (1 mg/mL) in 10% SDS-solution (w/v). Gel electrophoresis was carried out at 60 V for 3 h in an agarose gel 1%, (w/v) with Tris–acetate/EDTA (TAE) buffer (20 mL TAE stock solution: 242 g Tris, 100 mL EDTA (500 mM, pH 8), 57.1 mL acetic acid (99%); ad 980 mL H_2_O). Finally, the gel was stained with ethidium bromide (10 μg/mL) for 20 min and detected with a ChemiDoc XRS system (Biorad, München, Germany).

### Isolating in vivo complex of enzyme (ICE) assay

Isolating in vivo complex of enzyme (ICE) assay was performed with modifications as described previously by Esselen and co-workers (2009). For the ICE-assay, 3,000,000 HT29 cells were seeded in cell culture dishes and were cultivated for 72 h. Cells were incubated with nevadensin, dimethyl sulfoxide (DMSO, 1%) and CPT (25 µM) or ETP (25 µM) for 1 h under serum-free conditions. Medium was removed and cells were rinsed with phosphate-buffered saline (PBS; 25 mL stock solution: 4.2 g KH_2_PO_4_, 180 g NaCl, 8.18 g Na_2_HPO_4_, pH 7.4 in 1 L H_2_O; ad 475 mL H_2_O). After that, cells were lysed with TE buffer (10 mM Tris, pH 8.0; 1 mM EDTA), containing 1% *N*-laurylsarcosyl sodium salt (w/v). Cell lysate was layered onto a caesium chloride gradient with decreasing density from the bottom to the top. Polyallomer tubes (14 mL, SW40, Beckman Coulter GmbH, Krefeld, Germany) were centrifuged at 200,000 *g* for 24 h at 20 °C. Afterwards, gradient was fractionated (450 µL/fraction) from the top of the tube. DNA content of single fractions was determined with NanoDrop (PeqLab Biotechnologie GmbH, Erlangen, Germany) spectrophotometer at 260 nm. All DNA-rich fractions were combined and blotted onto a nitrocellulose membrane using a slot blotting apparatus (Minifold II, Whatman/Schleicher & Schuell, Dassel, Germany). Membrane was blocked with TBST buffer (200 mM Tris, pH 7.5; 1.37 M NaCl; 1% Tween 20 (v/v)) containing 5% low-fat powdered milk. Detection was carried out with a mouse monoclonal antibody, against TOPO I (100 kDa; dilution 1:100 (v/v)) (Santa Cruz Biotechnology Cat# sc-271285), which is linked to horseradish peroxidase (HRP), or TOPO IIα (170 kDa; dilution 1:100 (v/v)) (Santa Cruz Biotechnology Cat# sc-165986). TOPO IIα samples were incubated with secondary antibody m-IgGκ BP-HRP in a dilution of 1:1000 (v/v) (Santa Cruz Biotechnology Cat# sc-516102). Chemiluminescent signals were measured with the ChemiDoc XRS system (Biorad, München, Germany). ImageLab 5.0 (Biorad, München, Germany) was used for the analysis of the blots. Total DNA content of the fractions was related to the arbitrary light unit and plotted as test over control (T/C ([%])

### Single-cell gel electrophoresis (comet assay)

Comet assay was performed following the protocol of Gedik and co-workers (1998). Therefore, 500,000 HT29 cells were seeded in cell culture dishes and were cultivated for 72 h. After that, cells were incubated with nevadensin, NC and PC (100 µM) for 2 h. After that, incubation medium was removed, and cells were rinsed with PBS. After removal of cells with trypsin, cell viability was determined with trypan blue. Cell viability of the samples should be > 80%. A duplicate of each sample, which includes 45,000 cells, was centrifuged for 10 min at 440 *g* and 4 °C. Cell pellets were resuspended in low melting agarose and transferred to normal melting agarose-coated microscope slides. After drying, coverslips were removed, and samples were lysed (89 ml lysis stock solution: 2.5 M sodium chloride, 100 mM Na_2_EDTA, 10 mM Tris, 1% (w/v) *N*-laurylsarcosyl sodium salt, pH 10; 1 ml Triton X-100, 10 ml DMSO) overnight at 4 °C. For electrophoresis, DNA was equilibrated for 20 min in alkaline electrophoretic buffer at 4 °C. After equilibration, electrophoresis was performed at 25 V, with a constant current of 300 mA, for 20 min. Microscopy slides were washed three times with neutralisation buffer for 5 min at 4 °C. DNA was stained with ethidium bromide (0.01 mg/mL), and DNA strand breaks were detected with a fluorescence microscope (Carl Zeiss Microscopy GmbH, Göttingen, Germany). For this purpose, 2 × 50 cells per slide and concentration were analysed with the Comet Assay IV System software (Perceptive Instruments, Suffolk, UK). Tail intensity was expressed as DNA signals in the comet head compared to the signals in the comet tail. Slides were encoded to provide an objective analysis. Intensities of the tail were expressed as T/C ([%]).

### Resazurin reduction assay

Cytotoxicity was investigated with the resazurin reduction assay, which was performed according to protocol of O’Brien and co-workers (2000). For experimental procedure 35,000 or 17,500 HT29 cells, respectively, were grown in 48-well microtiter plates for 48 h. Subsequently, cells were incubated with nevadensin, NC and PC (200 µM) in cell culture medium for different time points (24 h and 48 h). After incubation, culture medium was removed, and cells were washed with PBS. After that, cells were incubated with resazurin solution (resazurin stock solution: 1.44 g KH_2_PO_4_, 9 g NaCl, 59.5 mg Na_2_HPO_4_ in 1 L bidest. H_2_O; 1:10 diluted in serum-free media) for 1 h at 37 °C. Fluorescence was measured at *λ*_ex_ = 544 nm and *λ*_em_ = 590 nm with an Infinite M200 PRO microplate reader (Tecan Group Ltd., Männedorf, Switzerland). The relative fluorescence intensity is proportional to the viability and was presented as test over control (T/C [%]).

### Cell cycle analysis

500,000 HT29 cells were seeded in cell culture dishes and were cultivated for 72 h to analyse cell cycle distribution. Subsequently, cells were incubated with 100 nM nocodazole for 24 h for synchronisation in G_2_/M phase. Cells were rinsed with PBS, and nevadensin, NC and CPT (PC, 50 µM) were incubated for 24 h. After that, cells were washed with PBS and were removed from culture dishes with accutase® (activity > 500 U/mL; Pan Biotech, Aidenbach, Germany). Samples were centrifuged for 5 min at 200*g*. Supernatants were removed, and cell pellets were resuspended with PBS containing 5% FCS (v/v). After a further centrifugation step, cell pellets were fixed with ice-cold ethanol (70%, v/v). On the day of measurement, samples were centrifuged again, and ethanol was removed. After a washing step with PBS, cell pellets were resuspended in 495 µL PBS and 5 µL RNase solution (1 mg/mL in PBS) was added to each sample. Cell suspensions were mixed with 5 µL propidium iodide (2.5 mg/mL) and incubated for 15 min on ice. The measurement was performed on the FC500 flow cytometer (Beckman Coulter, Krefeld, Germany) with a flow rate of 30 µL/min. Excitation was performed at 488 nm and emission was detected at 620 nm. Initially, cell duplicates and aggregates were excluded from the distribution of the phases, so that only single cells were presented in the respective histograms. The phases G_0_/G_1_, S, and G_2_/M are adapted to the solvent control and adopted for further samples. The distribution of the phases was set to 100% and expressed as T/C ([%]).

### Caspase-3, -8 and -9 activity assays

Apoptosis induction was determined by induction of caspase-3, -8 and -9 activity using the protocols previously described by Krug and co-workers (2018). Therefore, 900,000 HT29 cells were seeded in cell culture dishes and were cultivated for 72 h. Cells were incubated with nevadensin, NC and PC (1 µM) for 24 h. Cells were rinsed with PBS, and 120 µL ice-cold lysis buffer (10 mM TRIS, 100 mM NaCl, 1 mM EDTA, and 1% Triton-X-100 in water) was added to each sample. After 15 min of lysis, cells were scraped off and transferred to reaction tubes. Lysates were centrifuged for 10 min at 10,000* g* and 4 °C. 30 µL of supernatants was directly mixed with reaction solution, which consists of two parts of reaction buffer (50 mM PIPES, 12.7 mM EDTA, 8.1 mM CHAPS ad 100 mL with water, pH 7.4), three parts water, half a part caspase-substrates (1 mM Ac-DEVD-AFC for caspase-3, 1 mM Ac-IETD-AFC for caspase-8 and 1 mM Ac-LEHD-AFC, for caspase-9, respectively (Sigma-Aldrich GmbH, Seelze, Germany) and 0.05 parts 1 M DTT solution using black 96-well microtiter plates. Mixtures were incubated for 1 h at 37 °C under dark conditions and measured at λ_ex_= 405 nm and λ_em_= 520 nm with an Infinite M200 PRO microplate reader (Tecan Group Ltd., Männedorf, Switzerland). Results were expressed as µmol AFC/µg protein.

A bicinchoninic acid (BCA) assay kit (Sigma-Aldrich GmbH, Seelze, Germany) was performed to determine total protein concentrations using external standard calibration with BSA. 15 µL of each cell lysate was mixed with 200 µL BCA reagent (BCA solution with 4% copper sulfate, 50 + 1, (v/v)), in a 96-well microtiter plate. Plate was incubated 30 min at 37 °C and absorbance was measured with an Infinite M200 PRO microplate reader (Tecan Group Ltd., Männedorf, Switzerland) at 560 nm.

### Lactate dehydrogenase (LDH) leakage assay

Lactate dehydrogenase (LDH) leakage assay was performed according to the protocol of Krug and co-workers (2018). Incubation and sample preparation were performed as described for caspase assays. Besides cell lysate, incubation medium was also needed for the measurement. 15 µL of lysate and 40 µL of incubation medium were mixed with 200 µL buffer solution (100 mM HEPES, 10 mM sodium pyruvate and 0.21 mM NADH, at pH 7.0) in a 96-well microtiter plate and were incubated for 30 min at 37 °C. Absorption was measured every 2 min at 355 nm in an Infinite M200 PRO microplate reader (Tecan Group Ltd., Männedorf, Switzerland).

### Hoechst 33342-staining for nuclear apoptosis analysis

500,000 HT29 cells were seeded out in quadriPERM® plates including SuperFrost PLUS™ adhesion slides and were cultivated for 24 h. Afterwards, cells were incubated with nevadensin, PC (CPT; 0.5 µM) and NC for 24 h. The culture medium was removed, slides were washed two-times with PBS (1×) and fixed with ice-cold methanol for 2 h at − 20 °C. After removing methanol, the slides were air dried for 30 min and stained with 1 µg/mL Hoechst 33342 in staining buffer (20 mM Tris–HCl, pH7; 150 mM NaCl, pH 7) on a laboratory shaker for 1 h. Staining solution was removed and slides were washed three-times with washing solution (2 mM Cu_2_SO_4_, 0.2 M CaCl_2_; 2 M NaCl; 0.2% Tween-20; 50 mM citric acid) for 5 min. Slides were directly washed two-times with PBS (1×) as well as two-times with pure water. Cover glasses were fixed on slides by using mowiol®. Slides were dried overnight at room temperature and detected with fluorescence microscope (Carl Zeiss Microscopy GmbH, Göttingen, Germany) with a DAPI filter at λ_ex_ 350 nm/ λ_em_ 461 nm. For this purpose, 1000 nuclei per slide were counted for each sample whereby slides were encoded. The results were expressed as percentage of apoptotic cells related to total cell count. Staining solution as well as washing solution following the method of Ligasová and Koberna (2019).

### Ferrous ion oxidation xylenol orange (FOX) assay

FOX assay was performed in accordance with the protocol of Jiang and co-workers with slight modifications (Jiang et al. 1992). Nevadensin in a final concentration of 250 µM was incubated in a 6-well plate in serum-free culture medium. Two differnt experimental approches were incubated, with and without catalase addition (100 U/mL). Incubation was carried out under cell culture condition for 24 h as described in Sect. 2.2. At selected times (0.5, 1.0, 2.0, 24.0 h), 90 µL of medium was taken and mixed with 10 µL MeOH in a reaction tube. Thereafter, 900 µL of freshly prepared FOX reagent ((NH_4_)_2_FE^II^(SO_4_)_2_ 250 µM, H_2_SO_4_ 25 mM, xylenol orange tetrasodium salt 100 µM, and d-(–)-sorbitol 100 mM) were added and homogenised on a laboratory shaker. After 30 minutes of incubation, an aliquot of 200 µL was transferred into a 96-well plate. Absorption was measured at 550 nm in an Infinite M200 PRO microplate reader (Tecan Group Ltd., Männedorf, Switzerland). Hydrogen peroxide concentration was determined with an external calibration in a range between 0 and 60 µM H_2_O_2_.

### Statistics and graphical software

Statistical evaluation was carried by using one-way ANOVA, followed by Tukey as post-hoc test. The presented data are the mean ± standard deviation (SD) of at least more than three independent experiments. Data analysis and graphics were prepared by the OriginPro 9.7 Software (RRID:SCR_014212).

## Results and discussion

### DNA intercalation and minor groove binding

**Fig. 2:**
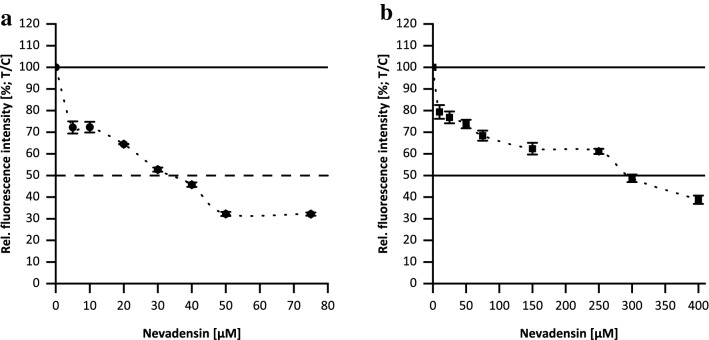
DNA-binding affinity of nevadensin investigated by the displacement of the minor groove binder Hoechst 33,258 (**a**) and the DNA intercalator EtBr (**b**). DMSO (1%) was used as negative control. The presented data are the mean ± SD of three independent experiments. The rel. fluorescence intensity was normalised to the NC and shown as test over control (T/C). The intersection point of the dashed line shows the half-maximum effect (IC_50_)

TOPO-poisoning effects of flavonoids are associated with their DNA-intercalating properties in literature (Snyder and Gillies 2002; Webb and Ebeler 2004; Bailly et al. 1999; Woynarowski et al. 1989). Therefore, at first the DNA-interacting properties of nevadensin were measured based on the replacement of the DNA intercalator ethidium bromide and the minor groove binder Hoechst 33258. Based on literature data for different flavonoids a relatively wide concentration range between 1 and 500 µM was chosen (Webb and Ebeler 2004). Nevadensin was incubated with double-stranded DNA, Hoechst 33258 or ethidium bromide and fluorescence was measured. The procedures were controlled by using the PCs netropsin (for Hoechst 33258; IC_50_: 0.31 µM ± 0.02 µM) and actinomycin D (for ethidium bromide displacement, IC_50_:1.93 µM ± 0.05 µM) (Supplementary Information Fig.S1). As demonstrated in Fig. 2a the intensity of fluorescence originating by Hoechst 33258 decreased with increasing concentrations of nevadensin (1–75 µM). The IC_50_-value of nevadensin was determined at 31.63 µM ± 0.96 µM. The ethidium bromide displacement assay resulted in an IC_50_ of 296.91 µM ± 19.32 µM (Fig. 2b). In sum, nevadensin was found to be more effective in Hoechst displacement assay and thus a higher affinity of nevadensin to the minor groove is consequently proposed.

In literature, the CPT derivative topotecan is also described to intercalate with DNA, whereas the underlying mode of action of CPT itself differs in the way of intercalation. CPT exhibits a poor or no binding to DNA or TOPO I alone, but it specifically trapped the DNA-TOPO I complex (Kerrigan and Pilch 2001). For the class of flavonoids, Webb and Ebeler (2004) suggest that DNA intercalation properties are not necessary, but sufficient for stabilisation of the cleavage complex. Their study provides structural alerts of flavones and flavanols, which are highly sufficient for DNA intercalators and TOPO I poisons. Nonetheless, some non-intercalating compounds are also capable to act as potent TOPO poisons (Webb and Ebeler 2004; Bailly et al. 1999).

Taken the DNA-binding properties of nevadensin into account the question arises whether nevadensin has also an impact on human topoisomerase in vitro.

### Impact of nevadensin on topoisomerase enzymes

**Fig. 3:**
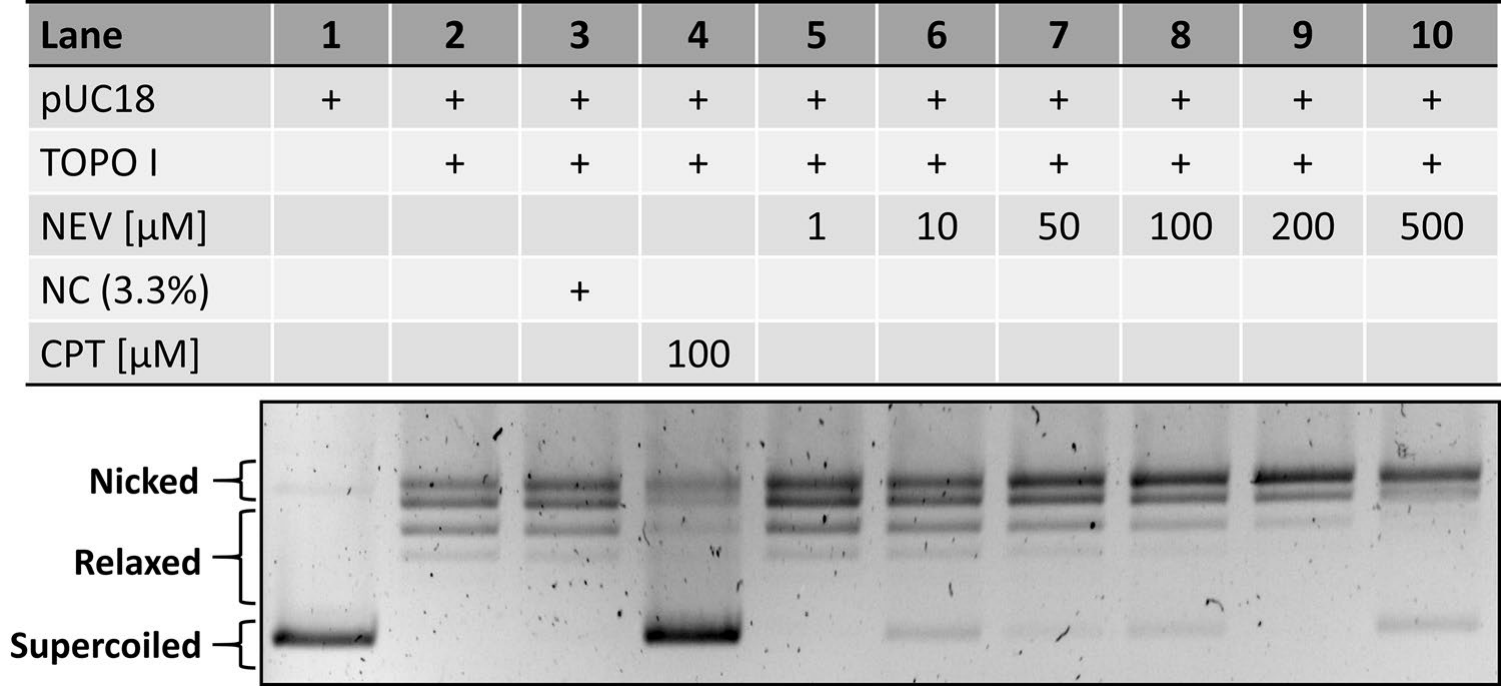
Inhibition of TOPO I activity under cell-free conditions induced by nevadensin in the relaxation assay. The activity of the enzyme was monitored by the transformation of supercoiled pUC18 DNA in its relaxed form. One representative gel out of at least three independent experiments is shown. Lane 1: supercoiled pUC18 DNA, lane 2: active TOPO I, lane 3: negative control DMSO (NC; 3.3%), lane 4: positive control camptothecin (CPT), increasing concentrations of nevadensin (NEV, 1–500 µM) (lane 5–lane 10)

The inhibitory potential of nevadensin on TOPO enzymes was investigated under cell-free conditions. First of all, the relaxation assay was performed to evaluate the impact on TOPO I. Active TOPO I enzymes catalyse the unwinding of supercoiled plasmid DNA (pUC18) and the relaxed DNA is separated by agarose gel electrophoresis. Results of the relaxation assay are presented in Fig. 3, including the supercoiled form of the used plasmid DNA (lane 1), active TOPO I (lane 2). NC (DMSO 3.33%, (lane 3), CPT (100 µM; lane 4) and of the test compound nevadensin in the respective concentrations (lane 5–lane 10). Nevadensin at concentrations > 100 µM led to a decreased formation of the relaxed DNA form in line with an increase of the nicked form (lanes 8, 9). At 500 µM nevadensin (lane 10) only intense signals for the supercoiled and the nicked DNA form were detected, suggesting a strong inhibitory effect.

**Fig. 4:**
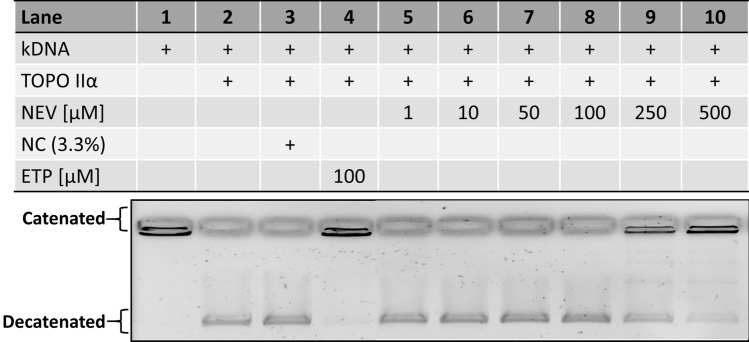
Inhibition of TOPO IIα activity under cell-free conditions induced by nevadensin in the decatenation assay. The activity of the enzyme was monitored by the transformation of kDNA into free mini circles. One representative gel out of at least three independent experiments is shown. Lane 1: kDNA, lane 2: active TOPO IIα, lane 3: negative control DMSO (NC; 3.3%), lane 4: positive control etoposide (ETP), increasing concentrations of nevadensin (NEV, 1–500 µM) (lane 5–lane 10)

Besides the effect on TOPO I the influence of nevadensin on TOPO II was also investigated with the decatenation assay. Results of the decatenation assay are presented in Fig. 4, including the catenated form of the kDNA (lane 1), active TOPO IIα (lane 2). NC (DMSO 3.33%, (lane 3), ETP (100 µM; lane 4) and of the test compound nevadensin in the respective concentrations (lane 5–lane 10). Nevadensin at concentrations 250 µM led to a decreased formation of the decatenated DNA form in line with an increase of the catenated DNA (lanes 8, 9). At 500 µM nevadensin (lane 10) the TOPO IIα activity is nearly completely inhibited, compared to the ETP. Consequently, nevadensin is suggested to act as TOPO I and TOPO IIα inhibitor, whereas the effect strength on TOPO IIα is more prominent under cell-free conditions.

**Fig. 5:**
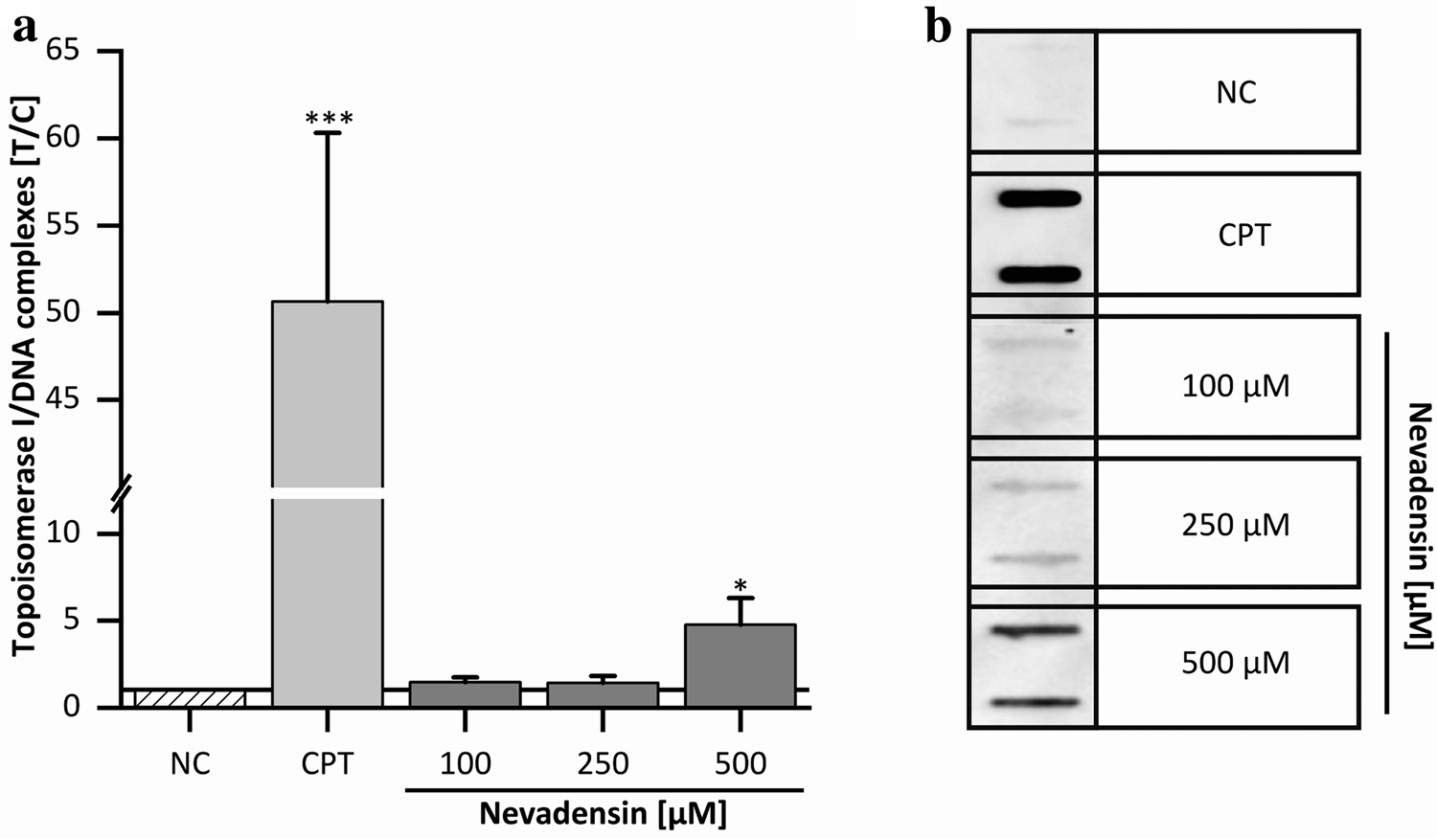
TOPO I-poisoning effects in HT29 cells after 1 h of incubation with nevadensin. Effects were determined by visualisation of TOPO I bound to DNA using an HRP-linked antibody against TOPO I. DMSO (1%) served as NC and CPT (25 µM,) as PC. Nevadensin was tested at a concentration range from 100 to 500 µM (**a**). The presented data are the mean ± SD of at least three independent experiments measured as duplicates. The amount of TOPO I/DNA intermediates was calculated as test over control (*T*/*C*) in relation to DNA content. Significances levels were determined with one-way ANOVA (Tukey as post-hoc test) and refer to the lowest concentration of 100 µM (*p < 0.05, ***p < 0.001). A representative immune blot is shown in (**b**)

Based on the above-mentioned results, the mode of action of nevadensin on TOPO I and TOPO IIα was characterised in more detail. Different modes of action of TOPO inhibition including catalytic inhibitors and TOPO poisons are described in literature. TOPO poisons stabilise the TOPO/DNA complex, the so-called cleavage complex. To clarify if nevadensin acts as a catalytic inhibitor or poison, the cellular-based ICE-assay was performed. After separation of DNA-bound TOPO from free TOPO protein, the amount of TOPO linked to DNA was chemoluminometrically analysed. As clearly shown in Fig. 5, the amount of DNA-bound TOPO I significantly increases at a concentration of 500 µM nevadensin. The lower concentrations also demonstrated a marginal increase but without but without significant relevance. However, no stabilising effects on TOPO IIα/DNA complexes were detected for nevadensin (Supplementary Information Fig. S2).

Concerning these findings, nevadensin was identified as selective TOPO I poison. Slight differences in the effective strength of nevadensin against the isolated and the cellular enzyme was found. Partial inhibition of enzyme activity was achieved at concentrations higher 100 µM in the cell-free assay. In contrast, significant TOPO I-poisoning effects were only detected at a concentration of 500 µM in HT29 cells. In comparison to the positive control CPT, the poisoning effect of nevadensin is noticeably lower. Interestingly, a total inhibition of TOPO IIα was also detected in the cell-free test system with isolated enzyme at 500 µM, whereas a poisoning effect was not verifiable in the cellular test system. Therefore, it is assumed that the concentration of 500 µM was not sufficient to interact with cellular TOPO IIα.

TOPO-poisoning effects of secondary plant constituents has been described for a wide spectrum of flavonoids. Effective concentrations are reported to be at the lower up to the moderate micromolar range in vitro. For example, fisetin (3,3′,4′,7-tetrahydroxyflavon) and quercetin (3,3′,4′,5,7-pentahydroxyflavon) achieved the half-minimal inhibitory concentrations (IC_50_ values) at approximately 71 µM and 42 µM in cell-free TOPO I assay (Constantinou et al. 1995). In the trapped in agarose DNA immunostaining (TARDIS) assay myricetin (3,3′,4′,5,5′,7-hexahydroxyflavone) showed TOPO I-poisoning effects at concentrations of approximately 111 µM (López-Lázaro et al. 2010). A few flavonoids, e.g., genistein (4′,5,7-trihydroxyisoflavone) act as TOPO II poisons. Bandele and Osherhoff demonstrated that myricetin acts as TOPO IIα poison in purified system but did not show any interaction with cellular TOPO IIα in ICE-assay, in line to the mentioned results with nevadensin (Bandele and Osheroff 2007), whereas investigation with TARDIS assay identified myricetin as prominent TOPO IIβ poison in human K562 leukaemia cells (López-Lázaro et al. 2010), indicating that the used test system and cell line could play a crucial role in identifying TOPO-poisons. Moreover, the used concentrations up to 500 µM of nevadensin could also be not sufficient to stabilise TOPO IIα/DNA complexes. In comparison, genistein showed effects at concentrations ≥ 100 µM in the ICE-assay (Kalfalah et al. 2011). The collective data corroborates that nevadensin seems to be a less potent TOPO-poison compared to other flavonoids. Of note, the cellular metabolism as well as cellular uptake of the compound might vary depending on the cell type and thus, might impair effect strength of each flavonoid at a cellular level. In this respect, it is also proposed that the chemical structure of nevadensin plays the most crucial role for its TOPO-inhibiting effects. Nevadensin only carries two hydroxy groups at position C-5 and C-7 and thus differs from the aforementioned members of the flavonoid category. The extent of hydroxylation might contribute to their respective effectiveness to affect TOPO activity, especially considering, e.g. hexahydroxyflavone myricetin as a reference. This hypothesis can be further supported by the two structural parameters, which have been defined by Bensasson and co-workers (2011) as follows: (1) Their three-dimensional structure attributed to their affinity to DNA and (2) the possibility of transformation into quinones by different oxidation pathways including autoxidation, enzymatic oxidation as well as formation of reactive oxygen species. It has been shown that the presence of further substituents at position C-2´and C-3 increases the dihedral angle O–2–1′–2′ between the chromone moiety and the phenyl ring these strongly influence DNA intercalation properties of flavonoids (Bensasson et al. 2011). Hydroxy groups at C-5 and C-4′ as well as the aromatic planar C-ring with the keto group at C-4 are further characterised as crucial features for potent TOPO I poisons. In the case of TOPO II-poisoning, the substitution pattern of the B-ring is already proved to be essential for poisoning effects. Moreover, it is figured out that the presence of additional hydroxy moieties at position C-3′ and/or C-5′ at the C-ring enhance the poisoning effect by a redox-dependent mode of action (Bandele and Osheroff 2007). The hydroxy moiety at position C-5, which is proposed to form a pseudo-ring with the aromatic, planar C-ring of flavanones bearing the ketone group at position C-4, allows the interaction between compound and enzyme (Bandele et al. 2008; Kozerski et al. 2003; Austin et al. 1992). These findings imply that the absence of hydroxy groups at positions C-3′ and C-5′ of flavonoids have a negative impact on TOPO II activity.

Besides aspects covering the cellular metabolism, the cellular uptake, and the structural diversity, H_2_O_2_ and ROS generation associated with flavonoids should be considered when investigating TOPO-poisoning effects. It is well known that the use of catalase reduces the formation of TOPO/DNA-complexes induced by (−)-epigallocatechin-3-gallate, gallic acid and myricetin in TRADIS assay indicating an impact of redox sensitive mechanisms (López-Lázaro et al. 2011). Most of the conducted studies with flavonoids were carried out without the application of catalase. Effects directly related to the compound could, therefore, not be distinguished from the secondary induction of H_2_O_2_ (Constantinou et al. 1995; López-Lázaro et al. 2010, 2011). This secondary effect might also be dependent on redox conditions applied in in vitro experiments and might not necessarily reflect the in vivo situation. In the presented experiments, the TOPO I poisoning effect of nevadensin is lower compared to the avaialble data for other flavonoids. Due to the use of catalse (see also 3.3.1), the direct impact of nevadensin plays the major role in the outcome under the conditions applied. However, the potential redox mechanism should be considered in follow-up studies to increase comparability between differnet flavonoids and to elucidate their lead mode of action in vitro/in vivo.

### Cellular response to TOPO I poisoning effects

#### DNA-damaging properties

**Fig. 6:**
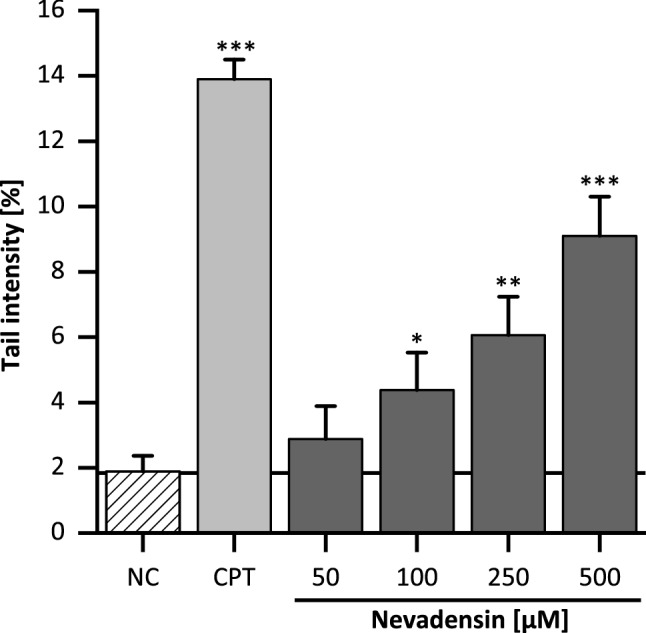
DNA strand-breaking properties of nevadensin in HT29 cells after 2 h of treatment investigated by the alkaline comet assay. DMSO (1%; NC) served as NC and CPT (100 µM) as PC. The presented data are the mean ± SD of three independent experiments performed in technical duplicates. Significances refer to the NC (DMSO 1%) and were determined with one-way ANOVA (Tukey as post-hoc test) (*p < 0.05, **p < 0.01, ***p < 0.001)

In literature, cleavable complex stabilisation and catalytic TOPO poisoning are associated with a reduced DNA integrity. Therefore, the DNA-damaging potential of nevadensin at concentrations of 50–500 µM was investigated in HT29 cells after two hours of incubation by using the alkaline comet assay. DMSO (1%) and CPT (100 µM) served as NC and PC, respectively. A concentration-dependent increase of DNA damage was detected after 2 h (Fig. 6). Compared to the known TOPO I poison CPT (PC), nevadensin demonstrated a slightly lower DNA-damaging effect. In comparison with other TOPO-targeting flavonoids such as the isoflavone genistein, which is described as TOPOIIα and TOPO IIβ poison (Schroeter et al. 2019), nevadensin demonstrated higher DNA-damaging potential in all tested concentrations. Secondary oxidative DNA-damaging properties of flavonoids contribute to the overall DNA strand breaks (Schroeter et al. 2019). Based on the use of catalase (100 U/mL) in each cell-based experiment an artificial formation of H_2_O_2_ released from nevadensin into the cell culture media was excluded. The results from the FOX-assay reveal that the use of catalase completely prevents H_2_O_2_-formation (Supplementary Information Table 1). Based on these findings in accordance with missing structural conditions of nevadensin to act as redox-sensitive TOPO-poison, further investigations on oxidative DNA damage were not performed. It can be mentioned that the observed TOPO-poisoning effect of nevadensin is strongly associated with an enhanced DNA damage. At concentrations ≥ 100 µM, a significant increase of DNA strand breaks was found. In the ICE assay an enhanced TOPO/DNA complex formation was detected, but the effect was only significant at the highest concentration of 500 µM. The stabilisation of cleavage complexes by TOPO poisons is a reversible process. It is assumed that in lower concentrations, an adequate time period of complex stabilisation is not warranted for a densitometric evaluation. However, the expected level of TOPO/DNA complexes might be likely sufficient to induce DNA damage. Nevertheless, it cannot be dismissed that the interaction with the minor groove and non-covalent association to DNA may also contribute to the formation of DNA strand breaks.

#### Influence on cell viability and induction of cell cycle arrest

**Fig. 7:**
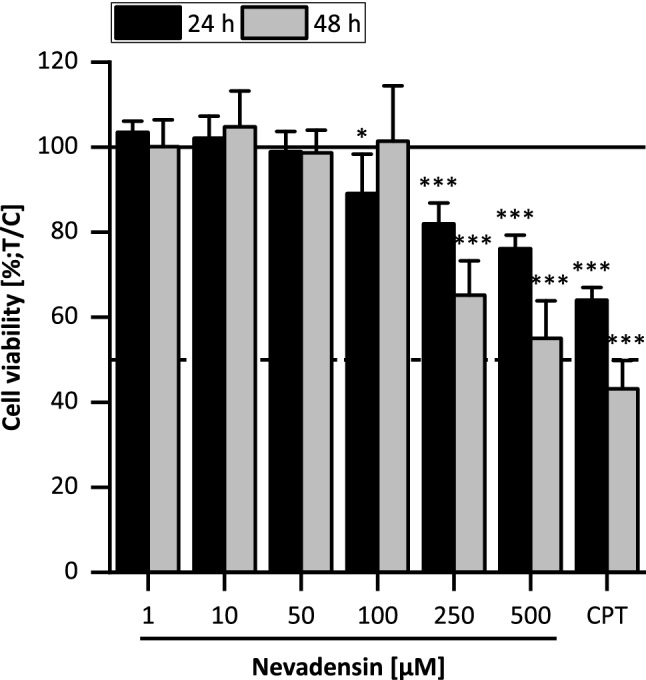
Cytotoxic effects in HT29 cells after 24 h and 48 h incubation with nevadensin investigated by the resazurin assay. DMSO (1%) served as NC and CPT (250 µM) as PC. The presented data are the mean ± SD of at least three independent experiments performed in a triplicate, calculated as test over control (DMSO = 100%). Significances were determined with one-way ANOVA (Tukey as post-hoc test) and refer to the lowest concentration of 1 µM (*p < 0.05, ***p < 0.001) for each time point

The functions of TOPO enzymes are described to be essential for replication and mitosis; hence, TOPO-inhibitors decrease cell viability and/or induce cell cycle alterations. Therefore, the impact of nevadensin on cell viability was investigated by using the resazurin reduction assay. The cells were incubated in a concentration range of 1–500 µM nevadensin for 24 h and 48 h, respectively. DMSO (1%) served as NC and CPT (200 µM) as PC (Fig. 7). After an incubation time of 24 h, a slight but significant reduction in cell viability about 20% was determined at nevadensin concentrations  ≥ 250 µM. After 48 h the cell viability was significantly diminished but still higher than 50%; thus an IC_50_ could not be calculated.

**Fig. 8:**
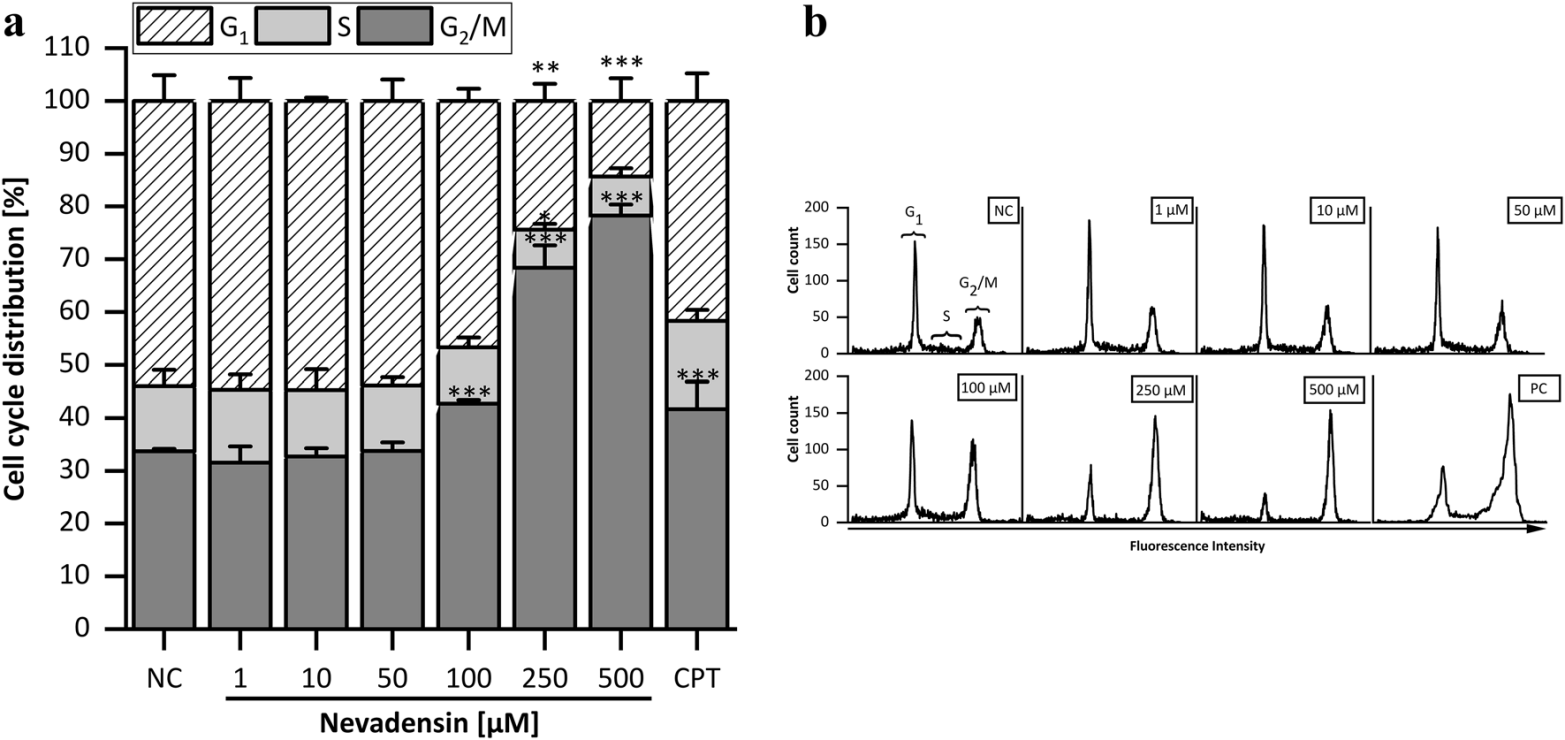
Cell cycle distribution of synchronised HT29 cells after 24 h of incubation with nevadensin (1–500 µM) (**a**). DMSO (1%) was used as negative control (NC) and 100 µM CPT as positive control. The data presented are the mean ± SD of three independent experiments. The significances were determined with one-way ANOVA (Tukey as post-hoc test) (**p < 0.01, ***p < 0.001) and refer to the negative control. Representative histograms of cell cycle analysis **(b)**

Besides the impact of nevadensin on cell viability, investigations on cell cycle distribution were also observed. Furthermore, TOPO poisons are reported to induce a cell cycle arrest (Kaufmann 1998). Therefore, HT29 cells were incubated with a non-cytotoxic concentration of nocodazole (100 nM) for 24 h for synchronisation in the G_2_/M phase (Supplementary Information Fig.S3) to ensure a coherent starting point for the subsequent compound treatment. Cells were incubated with nevadensin (1–500 µM) for 24 h. DMSO (1%) served as NC and CPT (50 µM) as PC (Fig. 8). An increase of cells in G_2_/M phase was found at nevadensin concentrations  ≥ 100 µM. The effect of 500 µM nevadensin on cell cycle distribution was considerably higher compared to CPT.

TOPO inhibition results inter alia in torsional stress during replication, which delays the transition of the cells from S to G_2_ phase. The subsequent cell cycle arrest is probably discussed as the result of the activation of the G_2_ control point. It is reported that the *G2/M* checkpoint prevents the division of cells with damaged DNA and, therefore, provides a chance to DNA repair processes (Löbrich and Jeggo 2007; Kaufmann 1998). In relation to the obtained marginal cytotoxicity of nevadensin, it is assumed that the strong G2 cell cycle arrest might initiate further cellular response mechanisms such as DNA repair. However, the decrease in cell viability gives a hint that cytotoxic events such as apoptosis or necrosis also occur as cellular response to nevadensin treatment.

#### Apoptotic or necrotic pathway

**Fig. 9:**
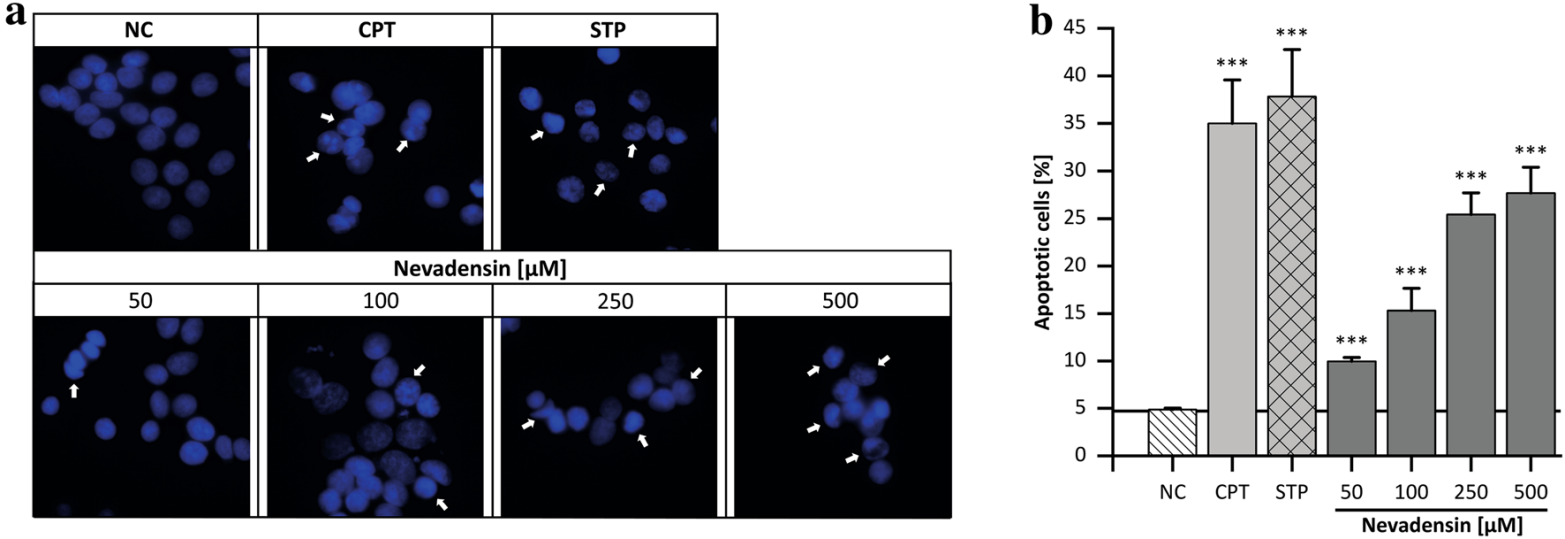
Cellular apoptosis observed with Hoechst 33342 staining. HT29 cells were treated with nevadensin (50–500 µM) for 24 h. DMSO (1%) was used as negative control (NC) and CPT (0.5 µM) as well as staurosporine (STP; 1 µM) were used as positive controls, respectively. Representative pictures are presented in (**a**), examples for apoptotic cells were marked with arrows. For the determination of the number of apoptotic cells, 1,000 nuclei were counted, and the rate of apoptotic cells are given in per cent. The data presented are the mean ± SD of three independent experiments (**b**). The significances were determined with one-way ANOVA (Tukey as post-hoc test) (**p < 0.01, ***p < 0.001) and refer to NC

**Fig. 10:**
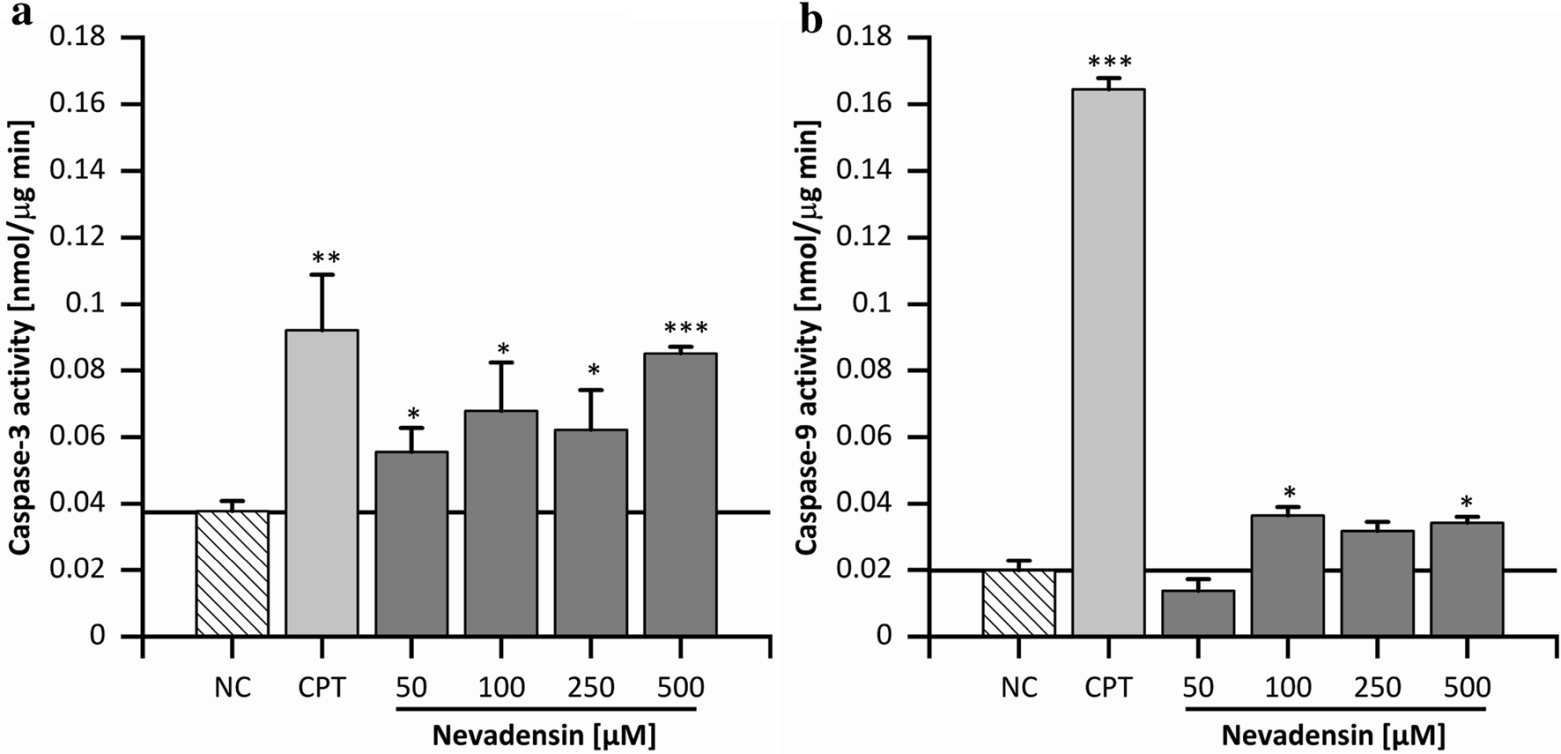
Casapse-3 (**a**) and caspase-9 (**b**) activation in HT29-cells after 24 h of incubation with nevadensin (50–500 µM). DMSO (1%) served as NC, CPT (1 µM) was used as PC. Caspase-3 and caspase-9 activation was analysed by using the specific substrates Ac-DEVD-AFC and Ac-LEHD-AFC. The presented data are the mean ± SD of three independent experiments. The significances were calculated with one-way ANOVA (Tukey as post-hoc test) (*p < 0.05, **p < 0.01, ***p < 0.001) and refer to the NC

For the investigations on cell death, the necrotic way of cell death was investigated, first. Therefore, the lactate dehydrogenase (LDH) leakage was used as marker for necrosis. HT29 cells were incubated for 24 h with nevadensin at concentrations of 50–500 µM because cytotoxic events occurred > 100 µM. LDH leakage was controlled with Triton X-100 as PC and DMSO (1%) as NC. After treatment of HT29 cells with nevadensin an increased LDH release to the medium was not detected (Supplementary Information Fig. S4). Consequently, it is excluded that nevadensin induces cell death via necrosis at the selected time. Based on these findings, the induction apoptosis was determined via Hoechst 33342 staining, first. In accordance with the cytotoxicity data an incubation time of 24 h was chosen. The assay was controlled by DMSO (1%) as NC and CPT (0.5 µM) as well as staurosporine (STP; 1 µM) as PC, respectively. Nevadensin demonstrated a concentration-dependent increase of apoptotic cells in HT29 cells after 24 h (Fig. 9b). In addition to the deformation and morphological changes of the nuclei, nevadensin also induced alterations in the chromatin structure (Fig. 9a). In addition, caspase-3 activity was also investigated after 24 h to further confirm the induction of apoptosis. The assay was controlled by CPT (1 µM) as PC and DMSO (1%) as NC. Nevadensin concentration-dependently increased caspase-3 activity and at 500 µM (Fig. 10a), a twofold, highly significant effect was found. TOPO-associated DNA damage is reported to modulate different apoptotic signalling elements (Sordet et al. 2003). For the mechanistic characterisation of apoptosis and for distinguishing intrinsic and extrinsic apoptosis, the activity of the effector caspase-8 and -9 was investigated. Caspase-8 was used as cellular marker for the extrinsic apoptotic pathway, whereas caspase-9 was implemented as an enzyme of extrinsic apoptosis. HT29 cells were treated using the parameters of the caspase 3 activity assay. Nevadensin significantly increased the activity of caspase-9 at concentrations > 50 µM (Fig. 10b). In contrast, no influence of nevadensin on caspase-8 activity was found (Supplementary Information Fig. S5). Compared to CPT an induction of both apoptotic pathways was excluded based on the negative caspase-8 assay. The results are in line with further studies, characterising TOPO poisons as primary inductors of intrinsic apoptosis (Abotaleb et al. 2018). Furthermore, this apoptotic pathway is highly associated with reduced DNA integrity being in accordance with the results of the comet assay (Ashe and Berry, 2003). Nevertheless, a G_2_/M arrest was observed after 24 h of incubation, thus supporting the assumption that nevadensin-induced DNA damage is recognised by cells and subsequent activation of control mechanisms leads to apoptosis and triggers cell viability. However, further mechanisms such as DNA repair are emphasised due to the fact that cell viability was only reduced up to 50% after 48 h of incubation. Intracellular uptake and metabolism, and further genotoxic properties of nevadensin might also influence these cellular targets and should be focused on further investigations.

Finally, the question raises about the relevance of TOPO-poisoning effects of nevadensin as well as subsequent cellular response in vitro in accordance to intake and bioavailability of dietary flavonoids in vivo. There is a leak of data regarding to dietary intake of nevadensin itself, up to now. However, it is known that the daily intake of flavonoids in Europe is about 428 ± 49 mg per day (Vogiatzoglou et al. 2015). Data on the bioavailability of nevadensin in human are equally unknown, so far. Based on animal studies, it is demonstrated that nevadensin is distributed rapidly and eliminated rather quickly. However, it is reported that nevadensin absorption is very poor after intragastric gavage whereas the absorption of aqueous solution is described as rapid, but its bioavailability is also low (Brahmachari 2010). The maximal plasma concentration of nevadensin in the rat was determined at 169 nM after single, oral administration of 50 mg/kg body weight (Liu et al. 2013). However, in the presented in vitro study micromolar concentrations were applied. It is concluded that systemic exposure after common dietary intake is unlikely to induce effects on TOPOs considering the available data basis. The systemic impact of dietary quantities of nevadensin on TOPO poisoning and its cellular effects is, therefore, rated as marginal. However, the local influence of the cellular uptake as well as the (cellular) metabolism in the gastrointestinal tract after dietary intake or in dosed form remains to be of scientific interest considering the very limited data situation in vivo.

Nevertheless, the presented in vitro study demonstrates mechanistical investigations on the impact of natural occurring food constituents on fundamental processes for the maintenance of DNA integrity and function is of utmost relevance for food safety.

## Conclusion

In this study, the impact of nevadensin on human TOPOs were investigated in cell-free and cell-based in vitro test systems in a concentration range of 1–500 µM. DNA-binding and intercalating properties were also examined because they are strongly associated with potent TOPO inhibitory properties. The investigations revealed that nevadensin has a high affinity to the minor groove of the DNA. In vitro models using isolated TOPOs characterised nevadensin as TOPO I and TOPO IIα inhibitor. The mode of action was further clarified in the in vivo complex of enzyme assay in the human colon carcinoma cell line HT29. Nevadensin induced a stabilisation of the cleavage complex, indicating a TOPO I-poisoning effect, whereas an effect on cellular TOPO IIα could not be verified in the tested concentration range. Furthermore, the study focused on the subsequent cellular response to TOPO I inhibition, whereby DNA-damaging effects, cytotoxicity, and cell cycle distribution were considered. It was found that nevadensin significantly induces DNA strand breaks resulting in a strong G_2_/M arrest. Additionally, investigations on cytotoxic effects demonstrated a significant decrease of cell viability after nevadensin treatment. Furthermore, the induction of apoptosis was proved by Hoechst 33,342 staining. The investigations on the mechanism of apoptosis induced by nevadensin indicated the intrinsic pathway, which is associated with caspase-9 and caspase-3 activation. Differences in effect concentrations between cell-free and cellular models as well as current limitations of cytotoxicity (about 60% viable cells) suggest that metabolism and cellular uptake as well as DNA repair mechanisms are potential further key pathways of the mode of action of nevadensin.

This study clearly highlights the versatile cellular mechanisms of food-borne flavonoids. The results provide first insights into potential adverse effects as well as favourable properties of nevadensin. Both should be investigated more precisely in vivo with specific regard to human exposure.

## Supplementary Information

Below is the link to the electronic supplementary material.

Supplementary file1 (DOCX 522 KB)

## Data Availability

All data generated or analysed during this study are included in this published article and its supplementary information files.

## Abbreviations

BCA: Bicinchoninic acid
BSA: Bovine serum albumin
CPT: Camptothecin
DMSO: Dimethyl sulfoxide
ETP: Etoposide
FCS: Fetal calf serum
FOX: Ferrous ion oxidation xylenol orange
HRP: Horseradish peroxidase
ICE: Isolating in vivo complex of enzyme
kDNA: Kinetoplast DNA
LDH: Lactate dehydrogenase
NADH: Nicotinamide adenine dinucleotide
NC: Negative control
PBS: Phosphate-buffered saline
PC: Positive control
ROS: Reactive oxygen species
STP: Staurosporine
TAE: Tris–acetate-EDTA
TARDIS: Trapped in agarose DNA immunostaining
TOPO: Topoisomerase
TOPO I: Topoisomerase I
TOPOII: Topoisomerase II
