# Supplementary material for: Topoisomerase poisoning by the flavonoid nevadensin triggers DNA damage and apoptosis in human colon carcinoma HT29 cells
Source: Arch Toxicol. 2021 Oct 12;95(12):3787–802. doi: 10.1007/s00204-021-03162-5 (PMC8536574; doi:10.1007/s00204-021-03162-5)
Supplement: Supplementary file 1 — Supplementary file1 (DOCX 522 KB) [file 204_2021_3162_MOESM1_ESM.docx]

**Supplementary Information**

**Topoisomerase poisoning by the flavonoid nevadensin triggers DNA damage and apoptosis in HT29 cells**

Lena Müller^1^, Larissa Rhonda Friederike^1^, David Bücksteeg^1^, Julian Alfke^1^, Thomas Uebel^1^, Melanie Esselen^1^

^1^University of Münster, Institute of Food Chemistry, Corrensstraße 45, 48149 Münster, Germany

*Corresponding author:

Professor Dr. Melanie Esselen

University of Münster

Institute of Food Chemistry

Corrensstraße 45

48149 Münster

Germany

E-Mail: esselen@uni-muenster.de

Journal name: Archives of Toxicology





**Fig.S1** DNA-binding affinity assay was controlled by netropsin for the displacement of the minor groove binder Hoechst 33258 **(a)** as well as with actinomycin D for the displacement of ethidium bromide (EtBr) **(b)**. DMSO (1%) was used as negative control. The presented data are the mean ± SD of three independent experiments. The relative fluorescence intensity was normalised to the negative control (DMSO) and shown as test over control (T/C; %). The intersection point of the dashed line shows the half-maximum effect (IC_50_)


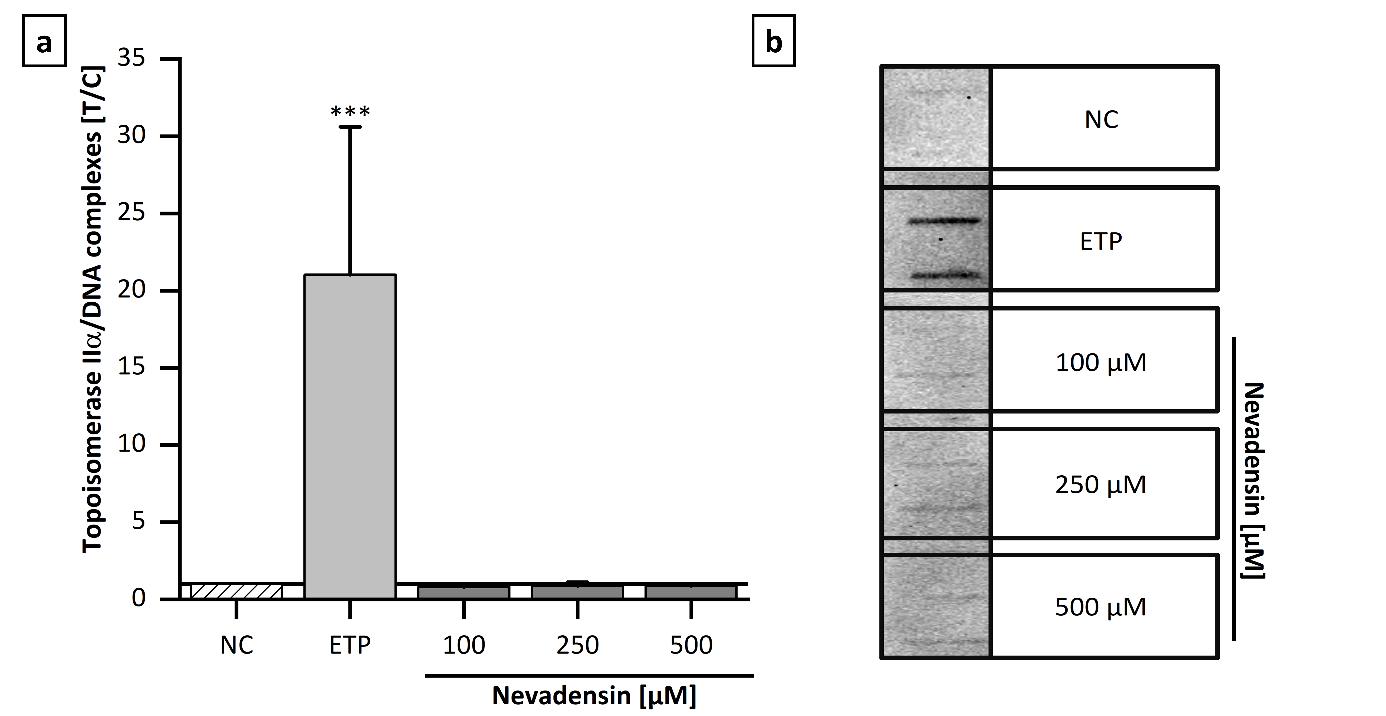


**Fig.S2** TOPO IIα-poisoning effects in HT29 cells after 1 h of incubation with nevadensin. Effects were determined by visualisation of TOPO ІIα bound to DNA using an HRP-linked antibody against TOPO I. DMSO (1%) served as NC and ETP (25 µM,) as PC. Nevadensin was tested at a concentration range from 100 – 500 µM **(a)**. The presented data are the mean ± SD of at least three independent experiments measured as duplicates. The amount of TOPO IIα/DNA intermediates was calculated as test over control (T/C) in relation to DNA content. Significances levels were determined with one-way ANOVA (Tukey as post-hoc test) and refer to the lowest concentration of 100 µM (*** = p < 0.001). A representative immune blot is shown in **(b)**

**Table 1** Measured hydrogen peroxide concentrations in the ferrous ion oxidation xylenol orange (FOX) assay after incubation of nevadensin (250 μM) in cell culture medium with or without catalase for 24 h. The presented data are the mean ± SD of three independent experiments. The hydrogen peroxide concentration was determined with external calibration **(**n.d.: not detectable, concentrations < 1 µM)

| **Time**  **[h]** | **Without catalase**  **100 U/mL**  **[µM]** | **With catalase**  **100 U/mL**  **[µM]** |
| --- | --- | --- |
| **0.5** | 1.22 ± 0.72 | n.d. |
| **1.0** | 4.95 ± 3.08 | n.d. |
| **2.0** | 9.37 ± 4.88 | n.d. |
| **4.0** | 11.09 ± 4.34 | n.d. |
| **24.0** | 12.67 ± 9.12 | n.d. |





**Fig.S3** Medium control (MC) and arrest control (ARC) of HT29 cells after synchronisation with nocodazole (100 nM) for 24 h **(a)**. The data presented are the mean ± SD from three independent experiments. The significances are determined with one-way ANOVA (Tukey as post-hoc test) (** = p < 0.01, *** = p < 0.001) and refer to medium control. Representative histograms of cell cycle analysis are shown in **(b)**





**Fig.S4** Effect on lactate dehydrogenase (LDH) release in HT29 cells after incubation with nevadensin for 24 h. DMSO (1 %) served as negative control (NC) and Triton X-100 (0.1%) as positive control. The LDH leakage is determined as test over control (DMSO = 100%). The presented data are the mean ± SD at least three independent experiments. Significance levels are determined with one-way ANOVA (Tukey as post-hoc test) and refer to the lowest concentration of 50 µM nevadensin (* = p < 0.05)

**Fig.S5** Casapse-8 activity of HT29-cells after 24 h of incubation with nevadensin (50 µM – 500 µM). DMSO (1%) served as negative control (NC), camptothecin (CPT; 1 µM) was used as positive control (PC). Caspase-8 activation was analysed by using the substrate Ac-IETD-AFC. The presented data are the mean ± SD of three independent experiments. The significances are determined with one-way ANOVA (Tukey as post-hoc test) (** = p < 0.01) and refer to medium control
